# GRAPHIE: graph based histology image explorer

**DOI:** 10.1186/1471-2105-16-S11-S10

**Published:** 2015-08-13

**Authors:** Hao Ding, Chao Wang, Kun Huang, Raghu Machiraju

**Affiliations:** 1 Computer Science & Engineering Department, The Ohio State University, 43210 Columbus, OH, USA; 2 Biomedical Informatics Department, The Ohio State University, 43210 Columbus, OH, USA; 3 Electrical & Computer Engineering, 43210 Columbus, OH, USA

**Keywords:** phenotypical analysis, histology image exploration, visual analytics tool, graph visualization

## Abstract

**Background:**

Histology images comprise one of the important sources of knowledge for phenotyping studies in systems biology. However, the annotation and analyses of histological data have remained a manual, subjective and relatively low-throughput process.

**Results:**

We introduce Graph based Histology Image Explorer (GRAPHIE)-a visual analytics tool to explore, annotate and discover potential relationships in histology image collections within a biologically relevant context. The design of GRAPHIE is guided by domain experts' requirements and well-known InfoVis mantras. By representing each image with informative features and then subsequently visualizing the image collection with a graph, GRAPHIE allows users to effectively explore the image collection. The features were designed to capture localized morphological properties in the given tissue specimen. More importantly, users can perform feature selection in an interactive way to improve the visualization of the image collection and the overall annotation process. Finally, the annotation allows for a better prospective examination of datasets as demonstrated in the users study. Thus, our design of GRAPHIE allows for the users to navigate and explore large collections of histology image datasets.

**Conclusions:**

We demonstrated the usefulness of our visual analytics approach through two case studies. Both of the cases showed efficient annotation and analysis of histology image collection.

## Background

Large-scale phenotyping studies have recently gained considerable attention in biomedicine. Phenotypes occur in various forms and at different levels as enumerated in [1]. Histology images offer an important source of knowledge for phenotyping studies at the cellular and tissue levels. Pathologists have been traditionally cataloging and studying the morphology of cellular phenotypes arising from genomic alterations and adverse conditions [2].

Since the morphological phenotypes that manifest in histology images are highly heterogeneous and variable, the analyses and annotation of these images require well-trained experts (e.g. pathologists) and rely on specific abstract patterns that they glean through extensive experience. Still, there is much variability in annotations obtained from various experts, especially given the uncertainty that exists in the underlying biological mechanisms. With the development of high-content and high-throughput acquisition methods, there is an ever increasing availability of high-resolution digital histology images [3], requiring the use of effective tools to explore and annotate the entire dataset efficiently. It is, therefore, necessary to gain a global overview of the morphology while being able to discern differences between images from individual samples.

**Figure 1:**
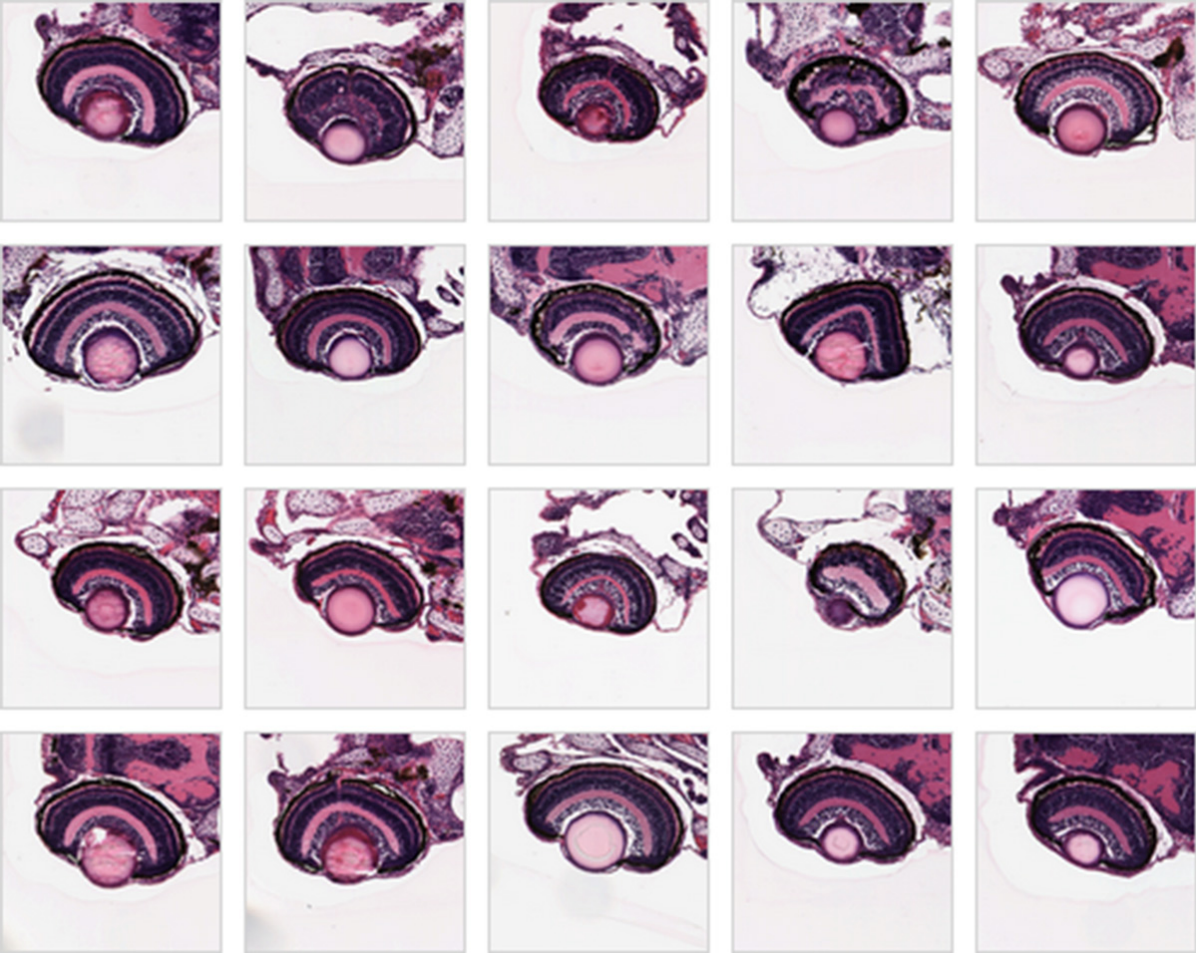
**Subset of histology image collection of zebrafish retinas arranged in a 2-D grid-layout**.

Consider the zebrafish histology image data subset presented in Figure 1. Each image depicts the retina of a zebrafish of a specific genotype. A typical phenotypical study involves differential comparison of these images to identify common and distinctive characteristics across the genotypes and identify the various subtypes in the collection [2]. A common approach towards the study and the annotation of images is realized by placing them side-by-side in a 2-D grid-layout (Figure 1). However, due to limitations in the human perceptual capacity, these approaches do not scale well. In addition, a 2-D grid-layout provides no information about how the images are similar or different, making it very difficult to learn the correlations across the image collection and to further annotate large repositories of data.

We believe that an interactive visualization tool that allows for the tangible organization of image collections can lead to a better overall understanding of the studied datasets as well as facilitate the image annotation processes. We present our visual analytics tool, **GRAP**h based **H**istology **I**mage **E**xplorer or GRAPHIE, designed to assist pathologists and systems biology researchers to explore, annotate and reveal potential relationships of phenotypical properties within a biologically relevant context. GRAPHIE employs the *bag-of-features *(BoFs) approach [4] to capture visual patterns from a given collection of histology images, thus allowing a semantic organization of unstructured image collections. By further using a proximity graph and a flexible graph layout, GRAPHIE provides a visual representation of the entire image collection, which in turn enables the intuitive exploration of the underlying structure of the dataset as well as the capability of drilling down to subgroups of interest. Our tool provides a rich set of interactive functions which make exploring and annotating on the graph more efficient and consistent. GRAPHIE also allows interactive feature selection, permitting users to examine subsets of image features and iteratively refine the graph visualization.

In summary, the main contributions of GRAPHIE include:

1 An interactive visual analytics tool to efficiently explore and annotate histology image datasets.

2 A framework which supports interactive comparison of subgroups and selection of features in histology images.

To evaluate GRAPHIE, we conducted two case studies. In the first study, we acquired histological images of 168 mutant specimens of the model organism *Danio rerio*, commonly known as the *zebrafish*. The mutant fish were the results of direct genetic alterations. GRAPHIE offers users a flexible way to explore distinctive morphological features and thus glean the structural changes wrought by genetic alterations. The second case study is concerned with histology images from a human breast cancer study. The task here is to explore image collections and annotate regions of the images if they predominantly contain specific tissue types (e.g., epithelium or stromal).

### Related work

Efforts have been expended to employ visual analytics approaches to explore image datasets. For instance, [5] visualizes an image collection using a multidimensional scaling layout based on semantic similarities between images. In [6] a method called clustered album thumbnails (CAT) was presented towards the hierarchical browsing of large image collections allowing users to interactively explore different levels of details. Both 2*D *grid and spiral layouts were used in [7] to present search results of images resembling an example image. While these methods support data exploration, they lack the ability to interactively assist users to spot differences among images, let alone glean subtle patterns in the more content-rich and complex histology image collection.

On the other hand, since manual annotation and analyzes of histology images requires well-honed expertise, the study of quantitative image-based assessment has attracted attention [8]. There is a considerable amount of work leveraging advances in the semantic content analysis to study histology images [9,3]. The SHIRAZ (System of Histological Image Retrieval and Annotation for Zoomorphology) project [2] proposed a content-based image retrieval system designed to rapidly annotate both histological phenotypes and identify potentially confounding imaging artifacts. In spite of their high performance, most of these automated systems do not allow users to incorporate domain knowledge in the annotation process. Our approach adopts semantic content analysis to facilitate exploration and annotation of the histology image collection. Additionally, GRAPHIE also allows the user to interactively evaluate the significance of image features.

Little work has been reported on the use of interactive visualization techniques to aid domain experts explore histology image collections. A web application for remote visualization and collaborative annotation of histology images was proposed in [10]. Jeong *et al*. introduced a visualization framework that targets interactive examination of histology image stacks [11]. While facilitating the annotation and analysis of histology images, these approaches still put the emphasis on individual images. In contrast, our approach systematically organizes image data and helps users discover implicit and latent relationships among phenotypes manifest as images.

There are many approaches to visualize the internal relationship of high-dimensional data. Techniques like the classic multidimensional scaling (MDS) and the heatmap have been used to visualize the similarity between images [12,5]. These techniques share the advantage of compactly displaying a large amount of data in an intuitive format. Our approach uses graphs to visualize the similarity structure of image collections. Graph representations are widely used in large population studies [13-16]. Interactive methods to explore and analyse network topology as well as the multivariate data are presented in [17]. In [18], Palmer argues that node-link representations are powerful to display the internal relationship. For this purpose, a graph has a more perceptual impact especially on gleaning proximal and similitude relationships [19]. Compared against the MDS technique, graph-based approaches maintain the topology and relationships among data entries, while, this information is difficult to glean after embedding in lower dimensional 2D planes or even 3D spaces. Additionally, GRAPHIE provides a much richer set of interactive analysis functions than the above approaches, allowing a more effective and efficient browsing of images. To the best of our knowledge, our prototype is the first system that allows users to focus on the entire histology image collection instead of an individual image.

## Task analysis and design

To better understand the needs in exploring histology data collections, we worked closely with domain experts. The domain experts expressed an interest in a visual analytics system to aid them in exploring and annotating histology images. Through our interactions with the analysts, we derived three main tasks that guided the design and implementation of GRAPHIE:

**Task 1 Overview of the histology image collection**. With a large histology image collection, it is important to gain an expedient overview, so that users can glean the similarities inherent in an image collection at a glance. Before analyzing an individual image, researchers often would like to have an overview of the entire image collection to learn the basic contents of a collection, their distributions, and their relationships at a glance.

**Task 2 Selection and comparison of images subgroups**. Selection is extremely important in an image-based phenotypical study. Our collaborators mentioned that during analysis that searching for images sharing the same morphological features and viewing images back-and-forth is time-consuming and tedious. Researchers seek more efficient ways to select desired images and scrutinize the morphological difference between subgroups of images.

**Task 3 Efficient annotation of images**. The annotation set is one of the key outputs of the histology image collection analysis. Our collaborators expect that the tool allows users to record the annotation while exploring the image collection.

GRAPHIE was designed to support these three tasks by providing a visualization overview of the image collection and an interactive user interface to examine individual images and the entire collection. Without such visualizations, the researchers' ability to explore and annotate the image collections is ineffective. After several iterations of discussions, we agreed upon using graph visualization to represent the image collection: that is each node in the graph represents an image and the layout of the graph reflects the multivariate distribution and depicts the similarity relationships present in the image collection. In addition, the selection and annotation of individual images can also be achieved by interacting with the graph visualization. We further justify the tasks and our approaches below.

### Image representation

In order to create a visualization of the histology image collection (**Task 1**), one first needs to define a way of measuring the similarity between images. Thus, it is necessary to use an image representation to summarize the images' content quantitatively. However, analyzing histology images is particularly challenging, since visual patterns are generally complex combinations of fundamental visual features associated with texture, color and shape [20]. Even with experienced pathologists, the visual inspection process often suffers from the disadvantages of being subjective, laborious, and insufficient when complex information is needed or is simply unknown. In this work, we employed the *bag-of-features *(BoFs) [4] approach to represent a collection of histology images. This approach is an evolution of texton-based representations and is also influenced by the bag-of-words representation for text classification and retrieval. Briefly, the BoFs approach works as follows: first sample images from the entire image collection are collected, image features are extracted from this subset, then a visual codebook is built as a summary of these feature vectors, finally, each image in the collection is represented by the frequency of the elements in the visual codebook that it contains. An important advantage of the BoFs approach is that it models image content in a robust way. The BoFs approach examines small characteristic image regions, allowing the representation of complex image contents without explicitly modeling objects and their relationships. In doing so, we simultaneously obviate the need for segmenting images-which is often in itself a formidable challenge. It should be noted that the BoFs approach has found much success when deployed on images and towards general computer vision tasks [4]. Further, the BoFs representation has been successfully applied to some problems in medical imaging [21].

### Graph visualization of image collection

The overview should display the image collection in a visually meaningful manner using sensible layouts (**Task 1**). In this work, we employ interactive graph visualization to provide an overview of the dataset and allow users to explore the entire image collection. In our case, the similarity between images is defined by the distance between corresponding image representations. The graph is constructed such that each node in the graph represents an image and nodes are only connected by an unweighted edge when two images are similar. Therefore, images with similar contents tend to be closer in the graph thus providing a convenient way to compare and analyze groups of subjects. There exist many similarity graph construction algorithms. In GRAPHIE, we choose *k*-Nearest Neighbor which has been widely used in many visualization and machine learning applications [22].

### Feature selection

Our collaborators observed that data-driven graphs may not capture the entire set of relationships of the target phenotype population. In other words, image representations generated in an unsupervised manner often contain a large amount of irrelevant and redundant features. This makes it very difficult to depict the true relationship between nodes in a given graph. For meaningful exploration, it is useful to find features that are highly correlated with distinct semantic classes. We have thus implemented a functionality via which a user can select arbitrary regions of the graph and examine which features can separate images into different classes. Users then can select the feature subset with higher distinctive power and use them to refine the graph visualization.

### Interactive user interface

Interactivity is an essential requirement given the complex nature of heterogeneity of histology image data. As stated in **Task 2**, the tool should allow users to select arbitrary subgroups in the graph visualization, and display them to help users to analyze differences or similarities among groups. We designed and implemented a web-based application with four interactive components, enabling flexible, efficient and adequate analytical functionalities for histology image data exploration.

### Image annotation

Scoring and annotating histology images plays an essential role in phenotype study (**Task 3**). The annotations provided by the pathologists could guide researchers to discover phenotype-relevant biomarkers [23]. Nevertheless, the annotated histology image collections are an important source of information and knowledge, which may support educational activities and various research studies. From a machine learning point of view, accurate annotations are considered to be valuable labels which directly influence the quality of automated histology image annotation systems. Given the complexity of visual patterns in histology images and the lack of rigorous phenotypical definitions, the traditional side-by-side manual annotation is often subjective, and sometimes an error-prone process [24]. Thus, the proposed tool allows users to interact with the constructed graph and enable the annotation of the image collection in a consistent fashion.

## GRAPHIE-Graph based Histology image Explorer

GRAPHIE is an interactive visual analytics tool designed for the exploratory analysis of histology image collections. We first describe the workflow that GRAPHIE realizes. Our workflow consists of two main parts:

1 **Back-end generation of image representation: **generate visual codebook and encode images as BoFs representations.

2 **Front-end visualization interface: **enable users to efficiently explore and annotate images collection by creating and manipulating a graph that accentuates similarities and distinctions across images.

### Back-end image representation generation

**Figure 2:**
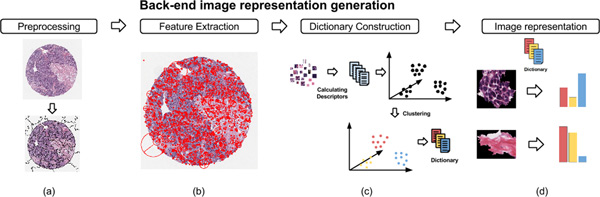
**Workflow to create image representations**: **(a) **Preprocess each given histology image. **(b) **Extract feature set. **(c) **Build a visual codebook through unsupervised clustering. **(d) **Generate the bag-of-features image representation

As we stated earlier, we choose the *bag-of-features *(BoFs) approach to represent a collection of histology images. This workflow of BoFs is summarized by the schematic in Figure 2. Now, we describe the specific steps of our image representation process:

#### Preprocessing

Generally, histology image data is replete with noise, artifacts and non-informative regions. Appropriate preprocessing of data is necessary for robust analysis. The choice of appropriate preprocessing methods will depend on the nature of the given image collection. In this work, the images were converted into gray scale and processed for noise removal using anisotropic diffusion filter [25].

#### Feature extraction

A scale-invariant feature transform (SIFT) [26] is employed towards the histology images in our study. SIFT descriptors are local and based on the appearance of objects or artifacts at particular interest points (e.g., scale-space extrema) and are invariant to image scale and rotation. They are also robust to changes in illumination and noise. Additionally, they are highly distinctive, relatively easy to extract and allow for robust object identification with a remarkably low probability of mismatch. Mikolajczyk and Schmid [27] suggested that SIFT-based descriptors tend to outperform the other descriptors (including Gabor filter banks, image moments, etc.) in many situations. Here we used the following parameter configurations to compute SIFT features: 8 orientations and 4 × 4 blocks of cells, resulting in a descriptor of 128 dimensions.

#### Visual codebook training

Given the feature descriptors extracted above, a visual codebook characterizing extant visual patterns is generated in an unsupervised manner. We use a clustering approach to prune down the data features to a core set of representative features (cluster centres) that constitute the visual codebook. The *k*-means algorithm is used in this work to find a set of cluster centroids that correspond to visual words. Although it has been reported that learning large numbers of *k *can improve supervised classification results [28,29], we observed that finding the appropriate number of *k *depends on the size of the image collection and the specific phenotypes present in the image collection.

#### Image representation

Given the visual codebook, a candidate image is represented as follows. Features are extracted from candidate image and then assigned to bins, where the bins are obtained by quantifying the feature space using the words in the codebook. The resulting histogram- where the count for each bin gives the frequency of occurrence of the words-is now the representation of the histology images.

### Front-end visualization interface

**Figure 3:**
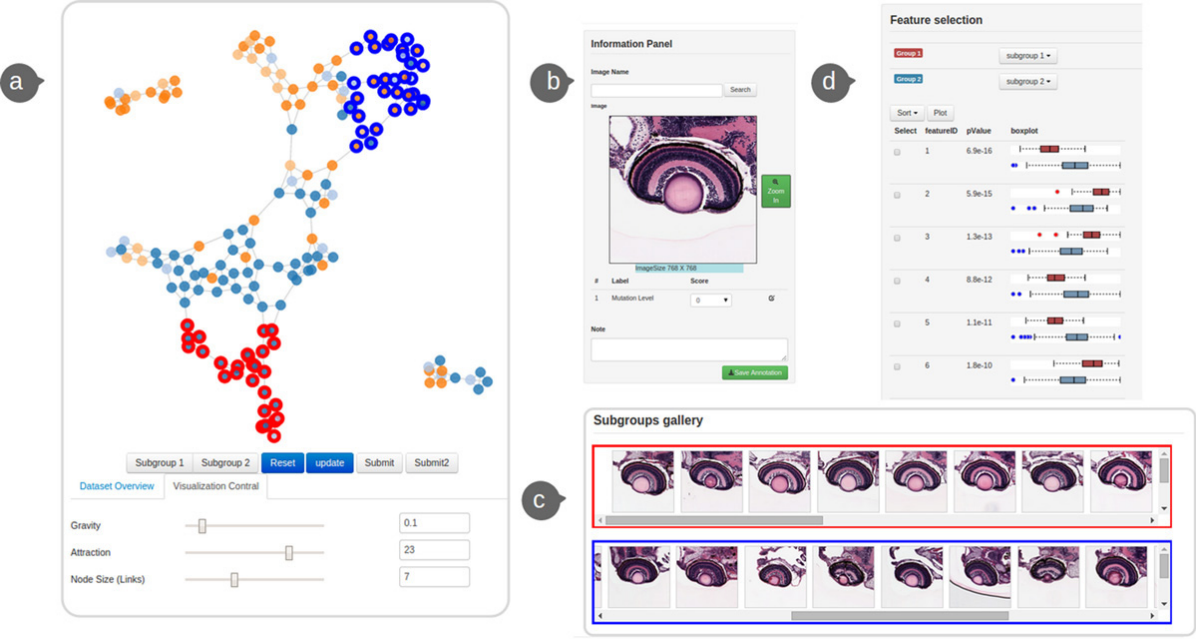
**The main GRAPHIE interface (a) **Graph visualization of the image collection from **case study 1**. **(b) **Individual image view. **(c) **Subgroups gallery. **(d) **Feature selection view. The user is comparing two subgroups of the image collection (red and blue rectangle in subgroups gallery) selected from the graph.

The design of GRAPHIE visualization interface follows the principles of creating efficient visualization systems suggested by well-known InfoVis mantras [30,31]. Particularly, we further deploy Shneiderman's general abstract tasks [30] to the following essential components. The front-end visualization interface consists of four main components: (a) Graph visualization of the image collection, (b) Individual image view, (c) Subgroups gallery, and (d) Feature selection view (Figure 3). Our interface uses multiple coordinated views [32] to visualize the given data collection. We aim to keep our user interface and interaction design as simple as possible.

#### Graph visualization of the image collection

With the BoFs histogram summarizing the content of images, all pairwise distances between images are computed using the BoFs histograms. Euclidean distance is used in this work. The resulting similarity matrix reveals the inner relationships of the entire image collection. The similarity matrix provides a fully connected graph, which is pruned down for effective visualization. The goal of pruning the graph is to extract and summarize the topology of the underlying feature space. As mentioned previously, GRAPHIE adopts the *k*-Nearest Neighbour (*k*-NN) to construct the unweighted graph. In a *k*-NN graph, each node only keeps edges which connect to the *k *closest nodes, where the euclidean distance between corresponding BoFs features defines closeness. Given the graph, we visualize it using force-directed graph layout-a method that has been shown to be effective in creating uncluttered visualizations [33]. The layout is fully interactive; users can adjust the graph layout by clicking and dragging the node. Users can also interactively browse a single image by clicking nodes to examine them in more detail in the individual image view. In order to avoid clutter, users can adjust the size of the nodes and the parameters pertaining to the metaphorical charges and forces that constitute the graph's layout. Nodes can be further colored with categorical annotations, thus allowing one to explore the structure of the data more easily. We provide more specific examples when we later discuss the two case studies.

#### Individual image view

The individual image view allows users to inspect individual images and annotation of them. Users can either select images by clicking nodes in the graph or by searching for them by name. This view also contains the preview and annotation of the selected image. Users can inspect the full resolution of selected images in a pop-up window by click the *zoom-in *button. There are two options to annotate the selected image: users can either select the corresponding score or qualifier by interacting with a drop down button or input notes in a text box at the bottom. Once, the entire image collection is annotated users could export the annotation by clicking the *save annotation *button.

#### Subgroups gallery

The subgroups gallery component enables browsing and comparing subgroups when needed. Each row in the subgroups gallery represents a subgroup of images. Users can select/update subgroups of the image collection by interacting with the derived graph visualization. Further, users can interactively browse the images by moving the scroll bar and clicking a thumbnail to examine each on the individual image view. The subgroups gallery also enables users to batch annotate all images in the subset by selecting the corresponding score with the drop down button on the left side.

#### Feature selection view

Although BoFs representation could effectively characterize the histology image, it is generated in an unsupervised manner and can inadvertently generate irrelevant phenotypical features. We implement the feature selection view which enables users to examine the distinctiveness of visual words for selected groups of images, therefore enhancing the visualization. Users can define the groups by selecting nodes from the graph visualization according to target phenotypes, or by using existing annotations. In order to select visual words that are most different between two groups of images, we conduct the Student's *t*-test for features to test hypotheses for group discrimination. We list features in ascending order using their *p*-values, computed according to Student's *t*-distribution. The features with more significant mean differences between selected groups are more likely to distinguish the images in those groups. Besides the significant statistic, we also provide boxplots for each feature to display summary information of feature distribution in selected groups. These plots help in better estimating the separability of the groups by the selected feature. In order to further investigate the quality of features, GRAPHIE enables users to select a feature subset from the given BoFs features. Then, users can regenerate the graph with the selected feature subset for the entire image collection to further inspect the changes in the new feature space.

### Implementation details

The visualization interface is developed in Javascript and *R *using the *R/Apache *module running on an *Apache *server. The data processing is implemented with *R *script which is triggered by Javascript. The interactive visualization is created using D3.js [34], a visualization JavaScript library.

## Results

In this section, we demonstrate the efficacy of our methods using two case studies. We described two repositories of data in our possession in the introductory section. The first case study serves the basic science community where the variety in histology of a specific animal model is examined. We especially focused on a portion of the zebrafish retina given that the structure is well understood and that we have labeled descriptions of the morphology. The second case study serves clinical practice where clinicians and pathologists classify patient biopsies to grade and diagnose diseases such as cancers. In this case study, epithelialstromal tissue slides were examined.

### Zebrafish retina histology images

In biomedical research, the use of animals such as zebrafish as a genetic model is useful for understanding the basic mechanisms that underlie human disease [35]. The heterogeneity expressed by the morphology in the eye of the zebrafish also makes the eye a perfect object for our phenotypical study. Like most classes of extant vertebrates, the retina of a zebrafish is composed of seven major cell types derived from the neural ectoderm, six types of neurons and one type of glial cell [36].

Our examples are obtained from the Zebrafish repository. A total of 168 images of larval zebrafish eyes were manually extracted from 20× magnification virtual slides acquired by the Zebrafish Functional Imaging Core facility at the Pennsylvania State College of Medicine. Image dimensions measure 768 × 768 pixels. Each slide is a well-stained with hematoxylin and eosin (H&E stains); the nuclei are the targets of hematoxylin while eosin stains the cytoplasmic and stromal regions. Each image is marked with a score (ranging from 0 to 3) which represent the level of phenotypical abnormality. A score of 0 indicates that the image has no visible abnormality while a score of 3 indicates an extremity of occurrence of corresponding abnormality. This ground truth scoring is manually recorded by pathologists with expert knowledge of the vertebrate anatomy. With the traditional slide-by-slide inspection, it is extremely difficult for a user to capture the subtle differences and annotate these images consistently and objectively. Even with the abnormality annotation made by a domain expert, it is still hard to comprehend the abstract visual pattern characteristics.

We now demonstrate how one can explore the zebrafish dataset with GRAPHIE. First the BoFs-based image representation was computed. In order to cover most of the visual patterns, 3 images were manually selected from each score subset (total 12 images) as representative images. We built the visual dictionary with features extracted these from 12 representative images. We tested with several visual dictionaries using *k*-means clustering, varying the value of *k*. The resulting graph visualization did not improve significantly when *k *given larger than 25, thus we chose *k *= 25 as the dictionary's size in this study. With the BoFs histograms, the similarity distance between images was calculated using the Euclidean distance metric.

**Figure 4:**
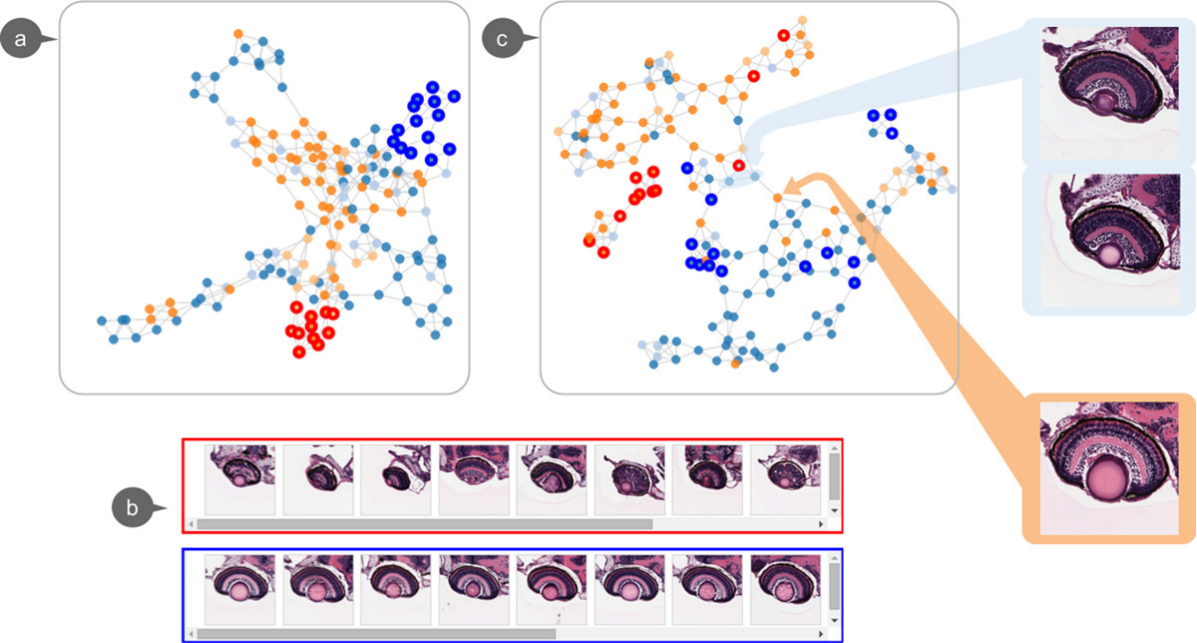
**Morphology zebrafish retina case study**. **(a) **Initial graph visualization. **(b) **Example phenotypes of the zebrafish retina: **(red) **retina of a mutant zebrafish. The mutant possess a small lens and fissure closure defect. **(blue) **Eye of wild-type zebrafish. Normal retinal lamination and a fused ventral fissure can be seen. **(c) **Graph created with selected features: the resulting graph shows better separation between wild-type and mutant-type zebrafish eye images, also helps users to spot the mis-annotated images. Here, we show three mis-annotated examples, with the upper two images were mis-annotated as wild-type and lower image being mis-annotated as mutant.

Figure 4(a) illustrates the initial graph visualization obtained in the form of a *k*-NN graph (*k *= 3) with 168 image nodes. The color for each node represents the abnormality score assigned to each image by our collaborator. The color blue indicates abnormality score of 0, which essentially implies that the genotype of the zebrafish as manifest in the image is wild-type. Light blue indicates abnormality score 1, a mutant zebrafish, with a subtle alteration from the wild-type phenotype. Light and dark orange respectively indicate that the mutant zebrafish has abnormality scores of 2 and 3, signifying a high level of abnormality in the specimen. By interactively examining the images in individual image view, we can observe that in the initial graph (Figure 4(a)), nodes close to each others tend to have similar properties and visual patterns. For instance, consider the nodes shown in Figure 4(b), the images lying in the red rectangle (nodes selected from bottom of the initial graph in Figure 4(a)) all have abnormality score 3. And in fact, the cells in these retinae are highly disorganized. On the other hand, the images in the blue box (images selected from upper right of the initial graph in Figure 4(a)) are resembling organizations often found in non-mutant, wild-type species. This distribution of nodes indicates that the proposed framework is able to capture different levels of phenotypical abnormalities present in the data.

However, the absolute separation between wild-type and mutant-type images remains unclear in the initial graph visualization. The small cluster in the upper left corner is connected to the main cluster of mutant-type images but is distinct from the main cluster of wild-type. Therefore, we performed feature selection in order to remove visual words that are irrelevant to the target groups. In this particular case, we used the abnormality annotations as the group labels. In order to pick the visual words that are most different between two groups of images, we conducted a Student's *t*-test on each visual word, computed per group to indicate the ability of separating the two groups. As the initial visualization is not optimal, a new graph is regenerated with the top 10 ranked visual words. As noted in Figure 4(c), the newly created graph has a much more clear separation between wild and mutant-types. We took a closer look at these images interspersed between distinct wild-type clusters. Interestingly, the abnormalities of these eyes are relatively subtle. When confirmed with our collaborators, some of these images were mis-annotated. With the graph visualization and subgroups gallery components, we could easily spot potential mis-annotated images which is a challenging task with the traditional 2-D grid-layout.

Through this case study, we can note that graph visualization is superior to a 2-D grid-layout for exploring image collections due to its ability to organize the images based on its content and reveal relationships between groups. By interacting with subgroups gallery and individual image view, one could efficiently compare and spot the differences between subgroups of images, therefore, form hypotheses regarding phenotypes. In addition, graph visualization can be improved by interactively selecting features with feature selection component.

### Epithelial-stromal tumor tissues

In a second case study, we deployed our methods on digitized tumor tissue slides. An important application of histology images is the examination of cancer tissue slides, wherein pathologists evaluate the composition of epithelial and stromal tissues and their interactions. Tissue slide analysis is the standard approach for the analysis of diagnostic, prognostic and predictive morphology biomarkers [37]. A majority of solid tumors are composed of epithelial cells. Classifying the tissue as epithelial or stromal is an important part of the automated cancer diagnosis [38]. In this case study, we demonstrate that through GRAPHIE researchers can gain a better perspective of the underlying structure of the tumor microenvironment that is composed of both epithelial and stromal tissues.

**Figure 5:**
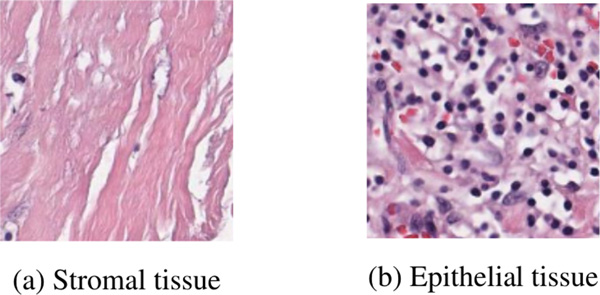
**Examples of heterogenous tissue compartments in the histopathological images of breast cancer in our adapted dataset**. **(a) **Epithelial tissue. **(b) **Stromal tissue.

Here, we present our visualization of the epithelial-stromal distributions. The histological images we used in this case study are collected from The Ohio State University (OSU) Pathology Core Facility. In order to view the datasets in more detail, we use the super-pixel method to divide each image into 50 patches [39] where each patch contains an approximately homogeneous visual pattern and homogeneous tissue (Figure 5a stromal / Figure 5b epithelium).

**Figure 6:**
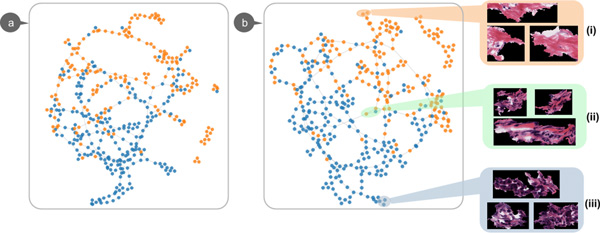
**Epithelial-stromal case study**. In graph visualizations, an orange-colored node represents a patch contains stromal; a blue-colored node represent a patch contains mainly epithelium. **(a) **Initial graph visualization. **(b) **Graph created with selected features.

The visualization workflow adopted here is similar to the one used in the previous case study. We randomly sample and select superpixel patches, and train a codebook. A superpixel patch represents a region of homogenous tissue. For each patch, we again generate a BoFs histogram to summarize the image. Here, each node represents a patch. Due to the large amount of nodes, clutter and costly computation are inevitable with the use of a straightforward force-directed layout scheme. To better interact with the visualization and to allow for the examination of the quality of visual words, we randomly sample 400 patches from the collection. In this case study, we carried out the following two explorations:

**Exploration 1: **Figure 6(a) is the resulting graph which consists of 400 nodes. The color of each node represents the type of tissue in that patch. A blue-colored node indicates a patch containing mainly epithelium; an orange-colored node indicates a patch that mainly contains stroma. We observe that the blue and orange nodes form two clusters. However, the graph visualization shows that clusters of mixed tissue prevail. Investigating the content of patches, we find that these patches contain both stromal and epithelial tissue. This artifact occurs because of the lack of quantitative control and subjective bias in our own manual labeling process.

**Exploration 2: **In another exploration, we perform the feature selection over the samples and create a new graph (Figure 6(b)) by using only the top 6 visual words. Comparing with the initial graph, the new graph shows an equally good separation between the two types of tissue patches with less visual words. Thus, the classification task can be efficiently achieved with a much lower feature dimensional space. It should be further noted that distinct groups are delineated. For instance, subgroup **i **(Figure 6(b)) includes purely stromal patches while subgroup **iii **includes only epithelium patches. In addition, subgroup **ii **includes patches with a mixture of stromal and epithelium.

## Discussion and conclusions

In this paper, we presented GRAPHIE, a visual analytics application designed to explore the histology image collection. By taking a data-driven approach, we developed an unbiased way for visualizing the entire collection. GRAPHIE not only provides an intuitive overview of the data but also enables users to use domain knowledge to improve the visualization through interactive feature selection. The visualizations and interactions of GRAPHIE are seamlessly integrated to allow users to effectively explore and annotate images, with a rich set of interactive functions. The use of GRAPHIE was evaluated with two datasets.

The current prototype implementation suffers from a lack-of-scalability, the size of the image collection that can be meaningfully explored is limited. We believed this problem can be tackled by applying different techniques pertaining to graph layout including semantic zooming, focus+context exploration techniques and sparse sampling strategies. In the future, we plan to test GRAPHIE with more histology image datasets and improve the image representation with more options of feature descriptors. We also aim to integrate with other types of data (e.g. genetic and epigenetic data) to enable an integrative phenotypical study. Finally, we plan to make our tool publicly available in the near future.

## Competing interests

The authors declare that they have no competing interests.

## Authors' contributions

HD carried out the system design and implementation, and drafted the manuscript. CW participated in the system design. KH and RM conceived of the study, designed the GRAPHIE, and contributed to discussions and suggestions to complete the manuscript. RM supervised the project. All authors read and approved the final manuscript.
